# Addition of fibers derived from paper mill sludge in paper coatings: impact on microstructure, surface and optical properties

**DOI:** 10.1038/s41598-023-46130-y

**Published:** 2023-11-07

**Authors:** Bilge Nazli Altay, Burak Aksoy, Anamika Huq, Richard Hailstone, Charles P. Klass, Muslum Demir, Scott Williams

**Affiliations:** 1 College of Engineering Technology, Print and Graphic Media Science, Rochester Institute of Technology, Rochester, NY 14623 USA; 2 Institute of Pure and Applied Sciences, Marmara University, 34722 Goztepe, Istanbul, Turkey; 3 Forest Products Development Center, College of Forestry, Wildlife and Environment, Auburn University, Auburn, AL 36849 USA; 4 Chester F. Carlson Center for Imaging Science, College of Science, Rochester Institute of Technology, Rochester, NY 14623 USA; 5 Klass Associates Inc., 118 131st Avenue East – Unit C, Madeira Beach, FL 33708-2628 USA; 6 Department of Chemical Engineering, Osmaniye Korkut Ata University, 80000 Osmaniye, Turkey; 7 School of Chemistry and Materials Sciences, Rochester Institute of Technology, Rochester, NY 14623 USA

**Keywords:** Environmental impact, Environmental economics, Forest ecology, Forestry, Ecology, Risk factors, Chemistry, Engineering, Materials science

## Abstract

Traditionally, cellulose nanofiber (CNF) production has primarily relied on virgin cellulose sources. Yet, the shift to using paper mill sludge (PMS) as a source for CNF underscores the significance of reusing and recycling industrial byproducts. PMS contains significant amounts of cellulose that can be extracted as a raw material. The purpose of present study is to provide a sustainable approach to PMS utilization as a paper coating additive in the cellulose nanofibrils (CNF_PMS_) form via simply scalable wire-wound rod coating method. The effect of CNF_PMS_ additive amounts at two coating layers on microstructure and surface properties of coatings such as porosity, air permeability surface roughness and optical properties such as brightness, gloss and CIE L*a*b* is studied, which they can also provide insight for the eventual print performance. Results indicated that the obtained CNF_PMS_ in paper coating shows 52% decrease in porosity, presenting significant improvement in the coating microstructure. The marginal increase in permeability coefficient and surface roughness, 54% and 10%, respectively, suggests improving color reproduction and preventing color density losses. Optical analysis showed slight decrease in brightness and gloss, as was expected. Notably, the lightness was improved, which also indicates increasing color gamut volume in printing applications. As a result, the current work offers a sustainable approach to manage PMS for use in paper coatings as a high-value-added material.

## Introduction

As the global economy grows, so does the rise in consumption and therefore the burden on natural resources. It is unsustainable for the planet to continue to consume its natural resources at the current rate, and therefore it is vital to the future of our planet and species to seek ways to conserve natural resources. Thus, sustainable development regarding the environment and natural resources has become an increasingly imperative issue globally. In a circular economy and strict legislative policies, it is essential to keep byproduct materials in circulation through restorative and regenerative methods to maintain their highest value for a long time^1,2^. Paper mill sludge (PMS) is a high-volume byproduct of the pulp and papermaking processes, and its disposal presents a significant challenge for waste management and a bottleneck for papermaking companies as the costs associated with PMS management and disposal on an economic, environmental, and social level are projected to rise in the future^3,4^. In comparison to current annual rates of 420 million (short) tons^5^, global paper and paperboard production is expected to increase to 550 million tons by 2050, boosting PMS production by 48–86%^4^. Typically, dry PMS is produced at a rate of 40–50 kg per ton of virgin papermaking (4–7% w/w of the PMS) and 300 kg per ton of recycled papermaking (20–40% w/w of the PMS)^6,7^. The pulp stock generated from paper recycling has shorter fibers, which leads to higher fiber loss on the pulp retaining sheet forming wire and increased PMS production^6^.

Even though PMS is often landfilled, creating environmental issues, it includes too many valuable cellulose fibers (11–76% w/w) to be considered as a true waste^8–10^. In the United States, for example, up to 87% of PMS was landfilled in 1979, although this proportion has since dropped to 52%^4^. Particularly in North America, PMS landfilling may be restricted or outlawed, which could result positively in a reduction in greenhouse gas emissions^4^. Thus, exploring PMS as a raw material in the extraction of cellulose might be a crucial strategy to reuse it in papermaking as well as other applications while contributing to the circular economy model. The new choices might also present chances for industrial synergies, which would be profitable for multiple parties. According to the idea of industrial ecology, the waste product of one industry becomes the raw material for another.

Beside fibers, PMS also includes filler minerals such as kaolin, CaCO_3_, silicate, TiO_2_ that are typical pigments used in papermaking and paper coating applications, pitch, lignin and heavy metals^11–14^. Other than landfilling, PMS is also used in low-value applications such as, farm fertilizing, contaminated soil remediating, animal bedding and incinerating^4,7^. The tonnages diverted to landfill sites may not be managed by existing energy recovery systems and land availability, depending on the local context for each paper mill^4^. The pulp and paper industry benefits from energy recovery through combustion (e.g., heat and power production), but it can be costly owing to the installation of combustion facilities and the necessary prior PMS dewatering process. Furthermore, in rare cases, the heavy metal content of some varieties of PMS and the odor, particularly when spreading near residential areas, might meet with environmental and public acceptability concerns^4^.

More recently, PMS has been reported to be utilized as a filler in the manufacturing of hardboard, fiberboard, cement, bricks, ceramics and concrete^7^. Other alternative uses of PMS includes fuel ethanol making^15^; nutrition rich material via vermicomposting^16,17^; catalyst for degradation of methylene blue^14^; adsorbent for the removal of hydrophobic substances^18,19^; and filler reinforcement material for composites where 40% increase was reported for bending modulus and 30% for bending strength^11,20^. However, the high moisture content of sludge (40 to 78% w/w) and increasing tipping fees make transporting PMS less economical. When PMS is transported more than 50 km from the mill for processing, dried-sludge is preferred over never-dried sludge for production^1,7^. Inappropriate drying on the other hand could adversely affect the functionalities due to hornification phenomenon, which is the formation of irreversible hydrogen bonding, van der Waals interactions, and covalent lactone bridges between cellulose fibrils during drying^21^.

In general, PMS is divided into two main types: low ash (< 30%, w/w) and high ash (> 30%, w/w) sludges in papermaking applications^7^, where the ash residue after high temperature removal of cellulose and other hydrocarbons is used to determine inorganic material content, mainly mineral pigments. The low ash sludges were reported to be obtained from tissue grade mills, groundwood and kraft mills, while high ash sludges were mostly generated from specialty grade printing and recycled paper mills related to the high level use of fillers in the papermaking processes^7,22^. On the one hand, higher-quality primary sludges have been found to have appropriate strength properties and are suitable for reuse in printing and writing papers, tissue, and wrapping papers applications. Upon bleach processing, secondary-quality sludges can be used in newsprint, magazine, and tissue applications. Without bleaching, they can be used for corrugated board, boxboard, and some tissue grades^8^. The tertiary-quality sludges on the other hand are appropriate for some packaging and construction-paper applications where the final product can tolerate a certain degree of dirt and contamination such as fluting and liner components^7,23^.

Recent research studies have been focusing on converting virgin cellulose fibers without having been subjected to any prior recycling processes into cellulose nanofibers (CNF) to produce high-value-added materials^24,25^. There are several methods for producing CNF, including chemical processing, mechanical disintegration processes such as homogenization, microfluidization, ultrafine friction grinding and sonication, or by acid hydrolysis with or without prior chemical or enzymatic pretreatment^25^. Due to the resultant CNF having high aspect ratio and large number of interfibril hydrogen bonds, the resultant product contributes to high strength and stiffness properties^26^. Other favorable properties of CNF count as low density, low coefficient of thermal expansion and moisture adsorption; higher thermal stability, surface smoothness, barrier properties and optical transparency^25,27^. To date, virgin fiber-CNF has been utilized in many applications such as papermaking^25,28^, 3D printing^29^, electronics^30,31^, smart wearables^32^, membrane in fuel cells^33^, tissue engineering^34^, adsorption^35^ and flame-retardant applications^36^. In papermaking applications, virgin cellulose fiber based CNF showed improved strength^37^, dry and wet web resistance to breakage^38,39^, retention of fillers including TiO_2_^38,39^, surface smoothness^28^, paper gloss^28,40^, oxygen permeability at high relative humidity (RH) values^41^, barrier properties^37,41,42^ and machine runnability^43^; while also decreased roughness^28^, porosity^37,41,43^, air permeability^37,38,41,42^ and dry pick resistance^42^.

As the reuse and revalorization of lignocellulosic wastes has becoming significantly urgent, PMS-derived CNF (CNF_PMS_) has been receiving more and more attention; however, the volume of research in this niche area is limited^44,45^. Traditionally, the production of CNF has been centered on using virgin cellulose sources. However, deriving CNF from PMS indicates a change in the origin of raw materials, highlighting the importance of repurposing and recycling industrial waste. The techniques employed to transform virgin cellulose into CNF can also be applied to produce CNF_PMS_. Nonetheless, particular research suggests that PMS can be fractionated into CNF_PMS_ without pretreatments because it already has short fibers and fines, leading to significant cost savings^8^. Previously, CNF_PMS_ was evaluated as a high-value material reinforcement agent^46,47^. When CNF_PMS_ was embedded into polyurethane (PU) at 1–4% ratio by weight to make composite film, tensile strength of the resulting composite was increased 24–160% and Young’s modulus value rise 90–350%, respectively, relative to pure PU^47^. One study concluded that samples made of 1% CNF_PMS_ in filter paper reduced paper opacity and tear index, while improving density, developing transparency, increased tensile strength and lower air permeance, which indicates lower porosity and a more compact network structure associated with the high specific surface area of CNF_PMS_ relative to virgin fiber-CNF^9^. The same study reported that a 93% reduction in total cost per ton can be achieved by using PMS to produce CNF, compared to using virgin fiber-CNF associated with high price sourcing and processing^8^.

**Figure 1:**
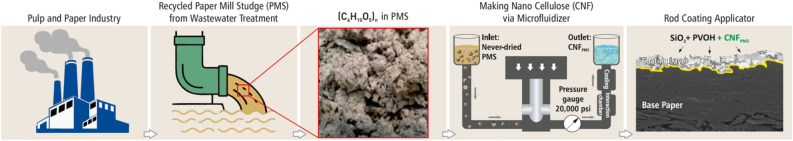
Schematic of PMS utilization for CNF making as paper coating additive.

The interaction between fibers, the properties of single fibers, and the chemical and mechanical structure of the coating layer applied to paper surfaces, all directly affect paper properties, including structural and optical properties of paper-based products^48^. Porosity and permeability are the structural properties of paper that influence ink penetration into the substrate. Porosity describes the fluid storage capacity, where low values indicate compact coating structure. The porous material's permeability refers to how easily fluids and gasses can pass through it. By using the values of porosity and paper thickness, the permeability coefficient can be estimated to rank porous media in terms of fluid absorption^49^. To establish the field further, the purpose of this research is twofold: to investigate the effect of two factors by conducting 4 × 2 factorial design analysis via a simple and scalable rod coating method: (1) CNF_PMS_ as additive (0, 1, 2 and 3 pph, where “pph” refers to the parts per hundred by weight added to 100 pph of the host material) and (2) coating layers (one vs. two layers) (Fig. 1). Changes in paper coating formulation are known to affect paper properties, so in this study, the optical properties (such as gloss and brightness) and structural properties (such as porosity, air permeability, thickness and surface strength) of the paper were analyzed as experiment responses.

## Materials and methods

### Microscopy and compositional analysis of PMS

**Figure 2:**
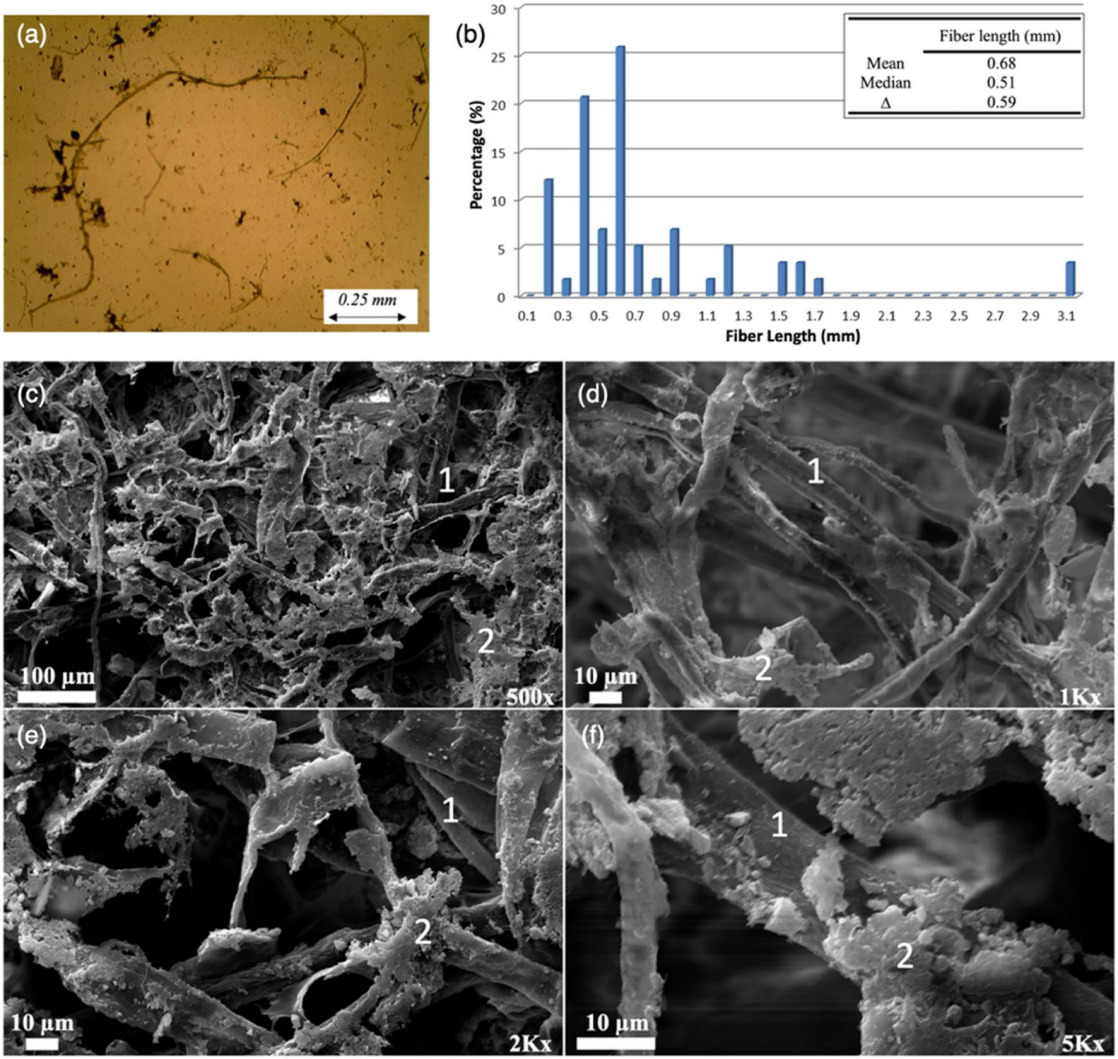
(**a**,**b**) Fiber length distribution of PMS before microfluidizer. Cellulose fibers (labeled 1), mineral particles (labeled 2) and other furnish components presented in SEM micrograph of PMS at (**c**) 500×, (**d**) 1 K×, (**e**) 2 K× and (**f**) 5 K×.

Never-dried PMS from a recycled papermaking process was provided by an undisclosed paper mill in USA. Compositional analysis was performed based on The National Renewable Energy Laboratory (NREL) procedures to determine carbohydrate, lignin, and ash content (Table 1) which revealed that the PMS is high-ash sludge (> 30% dry weight)^7^. The prepared samples were analyzed using a scanning electron microscope (SEM) (ZEISS DSM 940) equipped with an energy dispersive spectroscopy (EDS) detector was used to observe the surface morphologies of the PMS structures. Fiber length distribution was determined by using optical microscopy. Several photographs were taken of each sample at 10× magnification, and fibers were individually measured. A distribution was derived for a mean and a median fiber length (Fig. 2a,b). The morphology of the PMS was also captured using the SEM. In Fig. 2c–f, the entangled cellulose fibers, mineral particles and other furnishing components can be observed.

**Table 1:** Compositional and elemental analysis of PMS.

Compositional					Elemental		
Ash	Glucan	Lignin	Xylan	Mannan	Ca	Al	Si
40%	34%	13%	7%	1%	74%	12%	14%

### Preparation of CNF_PMS_

Microfluidizers require fiber lengths to be less than 0.3 mm for trouble free operation to produce CNF. For this reason, 24 g of recycled PMS fibers were refined using a PFI type refiner for 2 h at 10% consistency (solid content). Refined fibers were then diluted with water to 1% and then passed through a Britt Jar^50^, screen with 200 mesh (Ø = 76 µm) to separate fines under shear (750–2000 rpm). The fines that passed the Britt Jar screen were processed at the microfluidizer. The fines were passed through the narrow channels of the microfluidizer chamber (*M-110EH-30 from MICROFLUIDICS, Newton, MA, USA*)^25^ 10 times to produce CNF_PMS_ under pressure (20,000 psi) at a consistency between 0.5 and 1% as it comes from the Britt Jar.

### Coating formulation

Table 2 shows the coating formulation along with the factor and levels of the 4 × 2 factorial experiment design used for the study. All coatings additive components were based on 100 parts per hundred (pph) pigment, the host material—as defined earlier. The formulation included the fixed factors of silica fume as pigment, polyvinyl alcohol (PVOH) as binder and the controllable factor of CNF_PMS_ as an additive. The mixture was manually coated on the commercial copy paper surface as one and two layers using a wire-wound rod applicator of bar #15 (Article No. 2420 manual film applicator, BYK Instruments), where 15 defines the diameter in μm of the wire used for the windings around the core rod, and dried at 105 °C for 1 min via hot air dryer after each coating.

**Table 2:** Factorial design for the experiment.

Run #	Pigment (pph)	Binder (pph)	CNF_PMS_ (pph)	Coating Layer
1	100	20	0	1
2	100	20	0	2
3	100	20	1	1
4	100	20	1	2
5	100	20	2	1
6	100	20	2	2
7	100	20	3	1
8	100	20	3	2

### Coated paper characterization

Roughness and porosity values were measured with Parker Print-Surf (PPS) testing device (*TMI, Industrial Physics: New Castle, DE*). The PPS clamping pressure for roughness (soft backing) and porosity measurement was set to 1000 kPa. Paper thickness was measured using a digital paper thickness gauge (*TMI*). The permeability coefficient of coated samples was calculated using Eq. 1, where *K* is the permeability coefficient (μm^2^), *Q* is the Parker Print Surf flow rate (mL/min), and *L* is the thickness of the paper (m)^49^.

1 $$K = 0.048838 \times Q \times L$$

Brightness was measured according to TAPPI T-452 method with a Micro S-5 Brightimeter (*TECHNIDYNE: New Albany, IN*). Gloss was measured according to TAPPI T-480 method at 75° with a glossmeter (*TECHNIDYNE*). The paper color CIE L*a*b* was measured using a spectrodensitometer (*TECHKON: Danvers, MA*). L*, a* and b* represent lightness, spectral red-green region and blue-yellow regions, respectively.

### Coated paper SEM

Top down images of the samples were accomplished with a Mira field-emission SEM (*TESCAN USA, Inc.: Warrendale, PA*) operating at 10 kV. Prior to imaging the samples were sputter coated with a AuPd alloy. Ultra-smooth cross-sections were prepared with a LEICA ARTOS 3D ultramicrotome operating at − 120 °C using an FC7 low-temperature attachment. The samples were then coated with approximately 15 nm of carbon using an EMS 150 carbon coater to minimize charging in the SEM.

### Statistical analysis

The data were evaluated using a two-sample independent *t-*test using JMP Pro 16 (*JMP: Cary, NC)* statistical software to obtain *p* values. The null hypotheses for the study were whether the statistical mean of structural and optical property measurements has no effect with respect to the factors CNF_PMS_ addition and coating layers (${H}_{0}: {\mu }_{control}={\mu }_{experimental}$). The alternative hypotheses (${H}_{1}: {\mu }_{control}\ne {\mu }_{experimental}$) were the opposite of the null hypotheses. The significance level was set to α = 0.05 (95%). The null hypotheses were rejected when *p* value was less than 0.05.

## Results and discussion

### Microstructure and surface properties

**Figure 3:**
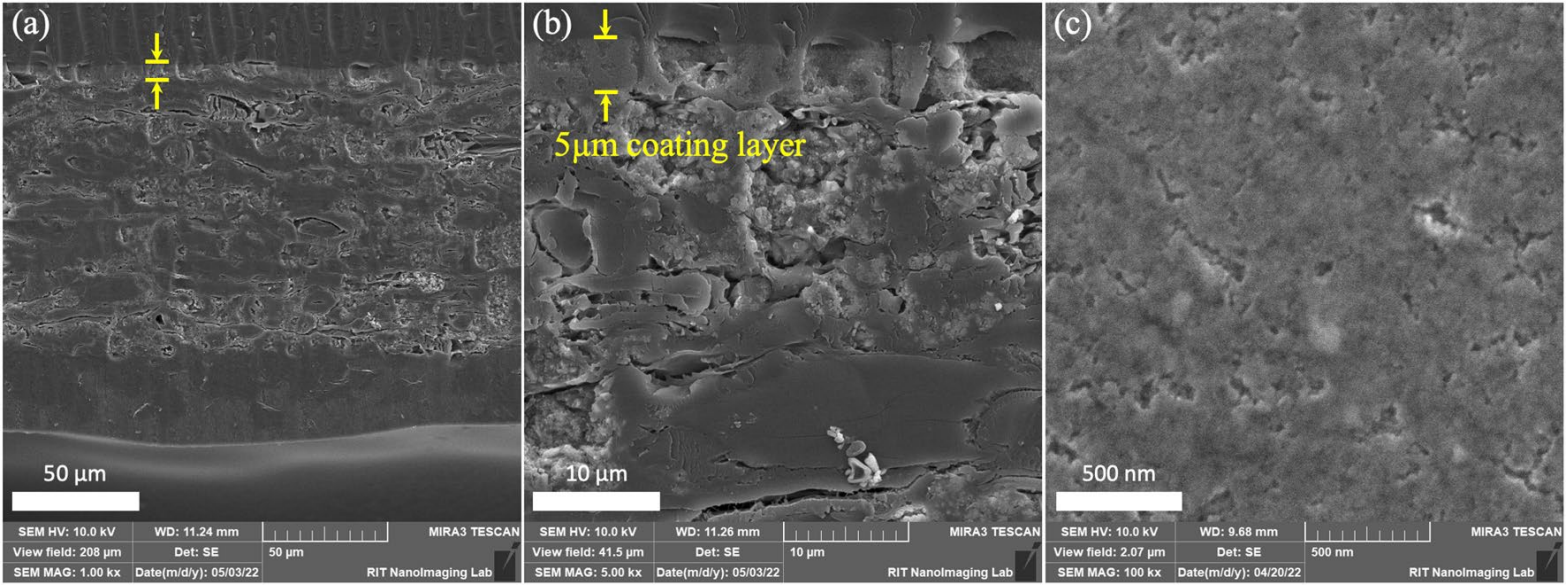
SEM micrographs of CNF_PMS_ coated paper (**a**,**b**) cross sections and (**c**) coating surface.

SEM images of the cross section (Fig. 3a,b) and coating surface (Fig. 3c) of the paper coated with CNF_PMS_ additive presents the representative coating layer measured as 5 μm. In general, small open spaces, voids and pores are distributed throughout the solid matrices in porous media such as paper and paperboard^48^. The dark spots in the cross section images of Fig. 3a,b presents the open space network that effect the adsorption and absorption of liquids.

**Figure 4:**
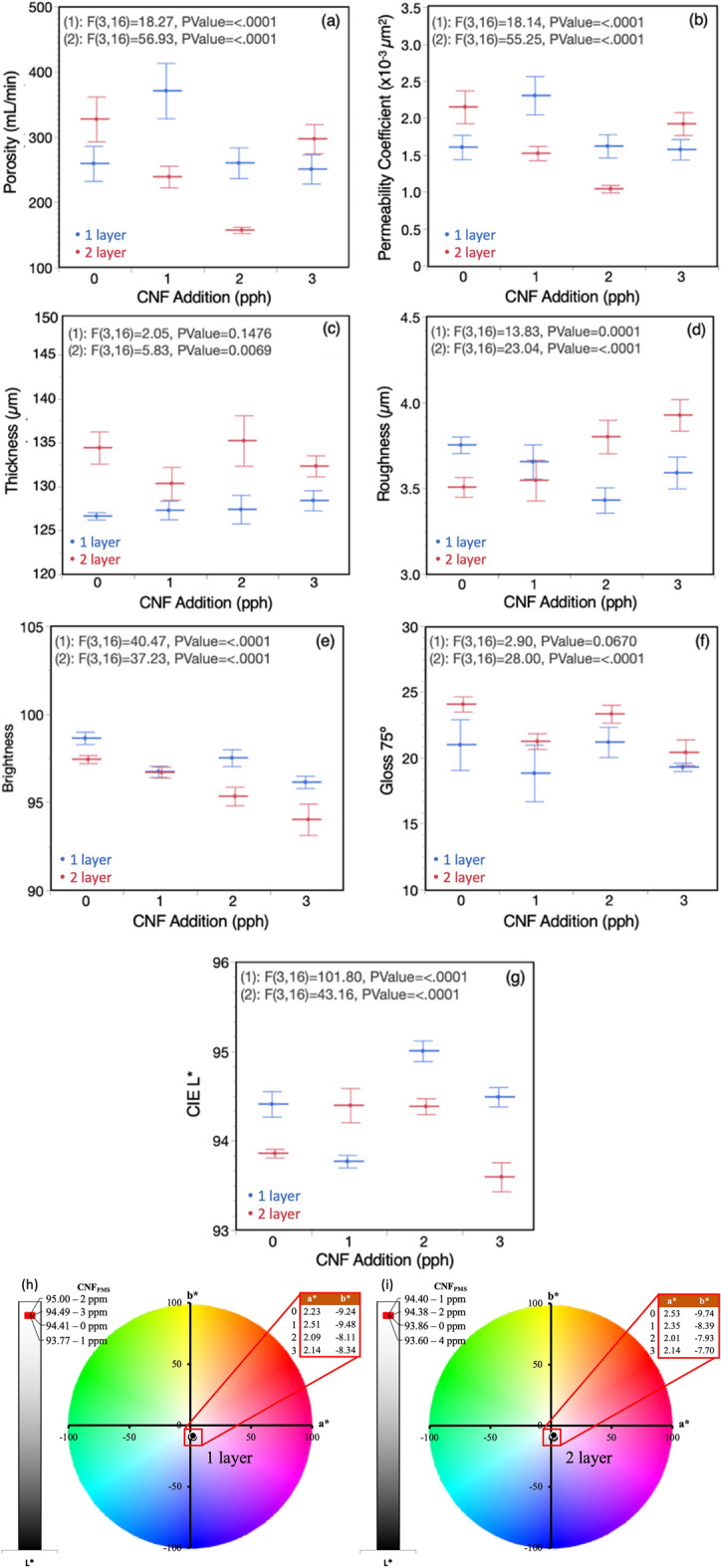
Structural (**a**–**d**) and optical (**e**–**i**) properties of CNF_PMS_ coated papers.

Papers are generally coated or laminated and sized with a variety of papermaking and coating materials to change the structural property of the interconnecting solid matrices to lower porosity and permeability^49^. Porosity describes the fluid storage capacity, where low values indicate compact coating structure. In previous studies, 17% reduction in porosity was reported by the addition of CNF derived from rice straw^43^ and about 33% by the addition of virgin fiber-based CNF at the ratio of 0.8 pph CNF to pigment^27^. In this study, porosity decreased 52% (from 328 to 158 mL/min) when 2 pph CNF_PMS_ was coated at two layers (Fig. 4a). The permeability of papers refers to how easily a fluid can pass through it under pressure. Using the porosity and paper thickness data, the permeability coefficient can be estimated to rank porous media in terms of fluid absorption^47^.

Figure 4b shows that the permeability coefficient decreased (from 2.2 to 1.0 × 10^–3^ μm^2^) as the CNF_PMS_ additive amount was increased in the formulation, in line with previous studies^37,38,41,42^. Similar to porosity, 50% reduction in permeability coefficient is realized. In general, a reduction in porosity and permeability improve color reproduction by allowing printing inks to stay on the paper surface since less ink penetrates the substrate and thus prevents color density losses^48^. The reason for the increase at 1 and 3 pph for porosity and permeability coefficient is considered here to be operational due to the coating application being manual.

Figure 4c presents that thickness for the first layer increased uniformly while the second layer had a decrease first, then increase followed by decrease again, presenting the common challenge of nonuniformity in lab drawdown application.

Figure 4d shows the surface roughness of paper being decreased as the CNF_PMS_ additive amount increased at one layer coating. The increase in roughness in the second coating layer is again thought to be caused by nonuniform manual lab drawdown. Overall, 7–52% decrease in porosity at one coating layer, and 31–35% decrease at two coating layers were observed with the CNF_PMS_ addition, indicating improvement in the microporosity of the coating layer. The roughness decreased 10% at each coating layer. Surface roughness is believed to affect ink transfer during the contact printing processes, such as offset lithography, gravure and flexography as the limited ink film thickness on the surface cannot reach deep pores^51^.

### Optical properties

The optical properties presented in Fig. 4e–i show the effect of the CNF_PMS_ coated papers on paper brightness, gloss, and CIE L*a*b* color coordination. In Europe, paper is categorized based on pulping method^7^, however, in the United States the fundamental criterion for the paper grade classification is the brightness, an arbitrarily defined parameter, of paper measured on a 0 to 100 scale, most papers having values in the 90 s. The sharpness of the text and images on a printed paper is believed to depend on how bright the page is^52^. In this study, the additions of CNF_PMS_ were seen to cause a decrease of 2–4 points in brightness (Fig. 4e) and 4 points in gloss (Fig. 4f). Light typically undergoes multiple scattering, reflection and refraction within paper causing an increase in brightness^52^. The compact network enabled by CNF_PMS_, evidenced by decrease in porosity, limits the scattering of light, thus the brightness decreases^52^. Although a statistically significant decrease was observed in brightness, it is still higher than the most common commercial paper grade brightness of 92. In general, two papers visually differing may have the same brightness value due to the brightness measurement, only focusing on one-third of the visible spectrum; thus, the only complete and absolute substrate characterization is by the three-number measurement CIE L*a*b* orthogonal system^52^. The effect of the CNF_PMS_ addition on CIE L*a*b* is presented in Fig. 4g–i, which indicates a significant increase in lightness was achieved with 2 pph CNF_PMS_ addition at one layer coating (Fig. 4g). The highest lightness suggests that a significant increase in color printing reproduction can be expected. Furthermore, the addition of CNF_PMS_ slightly moved color CIE a* and b* to greener (2.5–2.1) and yellower (− 9.7 to − 7.7) regions, respectively, toward a more neutral white (Fig. 4h,i).

**Figure 5:**
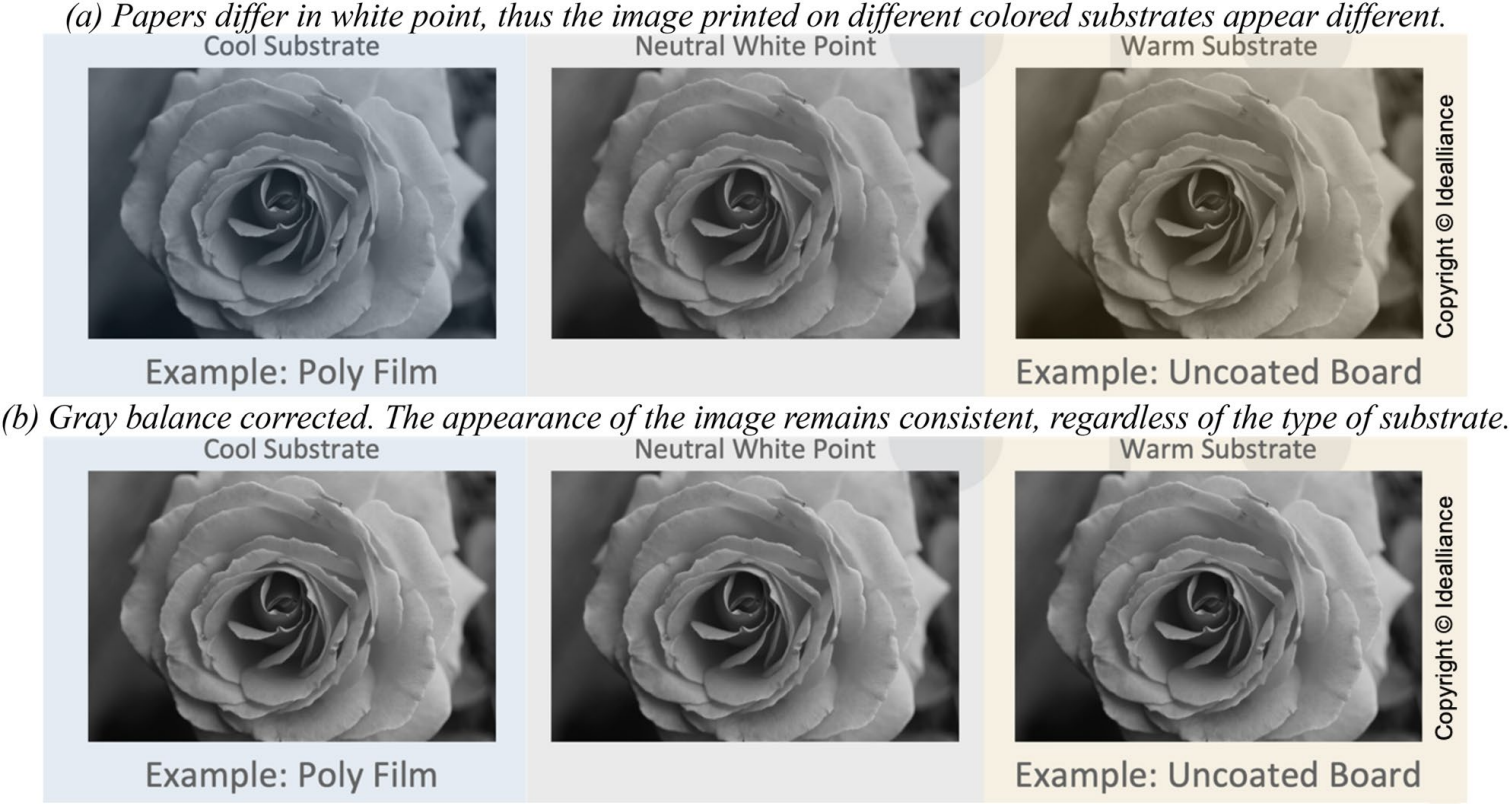
Image comparison on different substrates: (**a**) the image is affected by substrate color, (**b**) the image is not affected by substrate color thanks to the SCCA^53^.

Overall, the decrease in brightness and gloss may not be disadvantageous for the color printing reproduction, considering 35–50% beneficial decrease in porosity with the CNF_PMS_ utilization since the compact network enabled by CNF_PMS_ would allow ink pigments to sit on the surface and maintain higher density by lowering ink penetration into the coating structure. Moreover, the influence of paper color in the color printing applications is manageable by following procedures used for reproducing visually accurate colors, such as G7 (Copyright Idealliance and include G7 is a registered trademark of Idealliance), thanks to the grayscale colorimetric measurements between processes and/or various papers^53^. In general, papers differ in white point, thus images printed on different colored papers appear slightly different (Fig. 5a). Applying G7 calibration and process control methodology (Copyright Idealliance and include G7 is a registered trademark of Idealliance) and its Substrate Corrected Color Aim (SCCA) procedures, near neutral gray balance and common appearance on any substrate can be achieved (Fig. 5b)^54^.

Table 3 lists the optimal amount of CNF_PMS_ additive in paper coating that favors specific structural and optical properties. For instance, to improve porosity and permeability coefficient, the maximum decrease would be reached with an addition of 2 pph CNF_PMS_ at two coating layers. Minimum roughness or maximum lightness, on the other hand, would be reached with an addition of 2 pph CNF_PMS_ at one coating layer.

**Table 3:** Optimal amount of CNF_PMS_ additive.

Properties	Benefit^1^	CNF_PMS_ (pph)	Coating Layer	Outcome
Structural				
Porosity	↓	2	2	158
Permeability	↓	2	2	1.0
Thickness	=	1 and 2	1 and 2	127 and 135
Roughness	↓	2	1	3.4
Optical				
Brightness	=	2	1	98
Gloss	=	2	2	23.3
CIE L*^2^	↑	2	1	95.0

^1^↓, ↑ and = indicate the minimum, the maximum and no change, respectively.

^2^L* at these settings cause a* and b* to be 2.1 and − 8.1, respectively.

**Figure 6:**
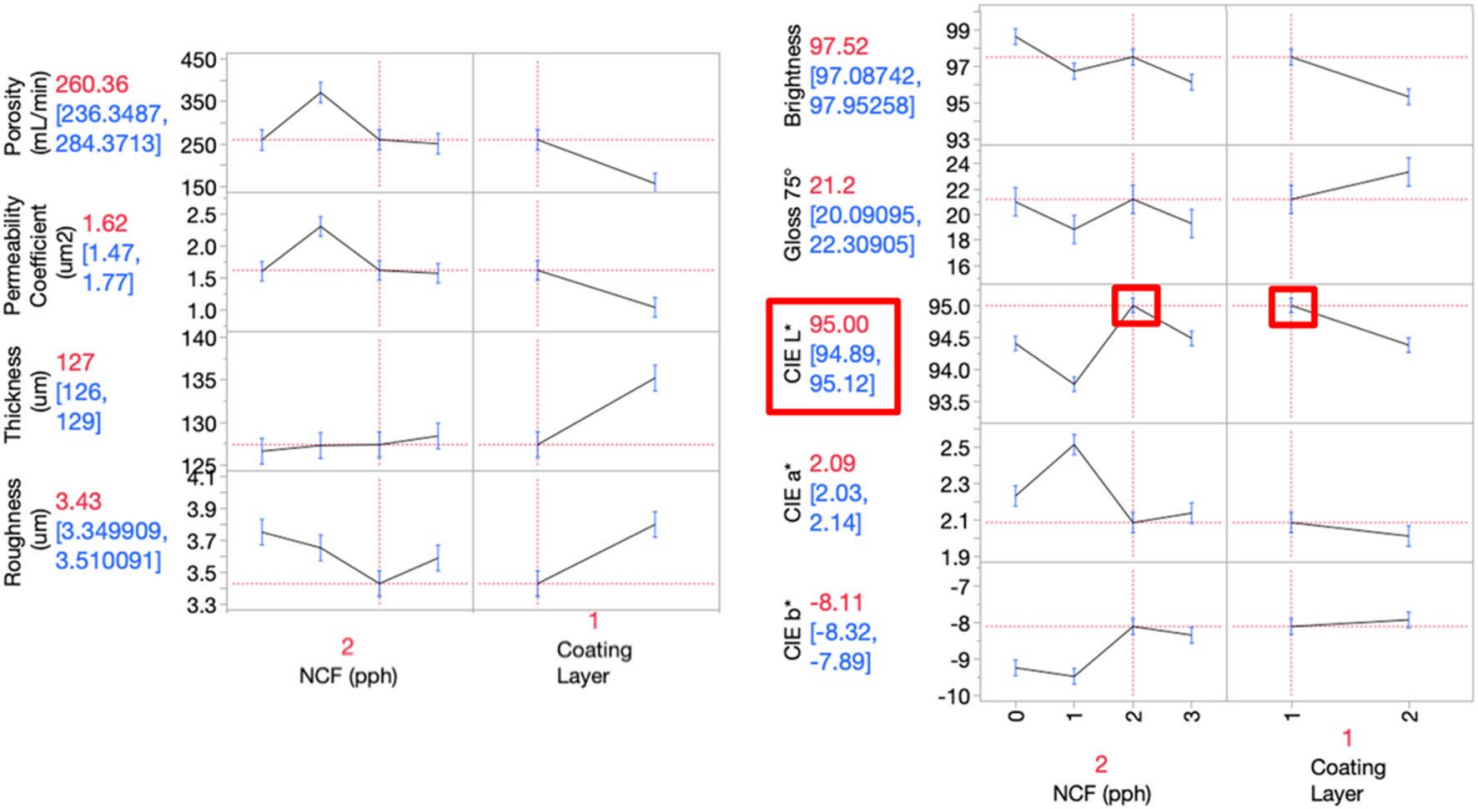
The predicted structural and optical properties based on the maximum CIE L* that positively benefit color printing reproduction.

Figure 6 shows the predicted structural and optical properties of CNF_PMS_ coated papers when the desire is set to achieve the highest CIE L* to benefit ideal color printing reproduction. A benefit of a factorial experimental design and resulting statistical analysis is the ability to fit a prediction model to the experimental results, where the model predicts to impact the individual factors have on the responses as well as potential interactions between the factors. Based on the prediction model fit to these experimental data, CIE L* is maximized at 2 pph CNF_PMS_ at one coating layer, resulting in an estimated average of 95 L*, 260 mL/min porosity, 1.6 × 10^–3^ μm^2^ permeability coefficient, 127 μm thickness, 3.4 μm roughness, 97.5% brightness and 21.2% gloss.

## Conclusion

Paper mill sludge (PMS), a high volume byproduct, contains cellulose fibers and fragmented fines up to 76%, making it a remarkable candidate as a raw material to extract cellulose. Cellulose nanofibers (CNF) produced from PMS via microfluidization process were utilized in a paper coating formulation as an additive. The results presented in this study shows the cellulose nanofibers production derived from PMS (CNF_PMS_) via microfluidization can be used as an additive in paper coating. The data showed that the additions of CNF_PMS_ decrease porosity, permeability and roughness of the paper significantly by improving the microporosity of the coating layer. The decrease in these structural properties would positively affect color reproduction by allowing less ink penetration into the substrate and suggests achieving high color gamut volume in printing. Slight decreases in paper brightness and gloss were observed, however they are negligible as the values were within the acceptable region prescribed by the paper industry. Also, G7*®* methodology can be adopted for substrate white point corrections. A significant increase in lightness (CIE L*) was observed in this study at 2 pph CNF_PMS_ addition at one layer, suggesting significant increase in color reproduction would be gained in printing applications. In the statistical software, prediction profiler model is used to observe multiple responses at the ideal setting of the highest CIE L* (2 pph, 1 coating layer) to understand the effect on the remaining structural and optical properties. In ongoing research, we are exploring printability on CNF_PMS_ coated papers using a standard color test chart for quantifying color gamut volume to validate the implications in this study.

## Data Availability

The datasets analyzed during the study are available from the corresponding author on reasonable request.
